# Lung function, allergic sensitization and asthma in school-aged children after viral-coinfection bronchiolitis

**DOI:** 10.1038/s41598-022-11356-9

**Published:** 2022-05-09

**Authors:** Sara Ruiz, Cristina Calvo, Francisco Pozo, Inmaculada Casas, María Luz García-García

**Affiliations:** 1 Pediatrics Department, Severo Ochoa University Hospital. Leganés, Madrid, Spain; 2 Alfonso X El Sabio University. Villanueva de La Cañada, Madrid, Spain; 3 Pediatrics Department, La Paz University Hospital, Madrid, Spain; 4 Translational Research Network in Pediatric Infectious Diseases (RITIP), Madrid, Spain; 5 TEDDY Network (European Network of Excellence for Pediatric Clinical Research), Madrid, Spain; 6 CIBERINFEC (ISCIII), Madrid, Spain; 7 Flu and Respiratory Virus Laboratory, National Center of Microbiology. ISCIII, Madrid, Spain

**Keywords:** Medical research, Risk factors, Signs and symptoms

## Abstract

Our main objective was to compare the lung function, the rate of allergic sensitization and the prevalence of asthma at 7–9 years in children hospitalized for bronchiolitis with viral coinfection versus single viral infection. Observational study in children with previous bronchiolitis and current age 7–9 years. Clinical data were collected. Fraction of exhaled nitric oxide (FeNO) determination, spirometry and skin prick test for common aeroallergens were performed. A total of 181 children hospitalized for bronchiolitis (40 coinfections and 141 single infections), with median age of 8.3 years (IQR:7.5–9.1) were included. Single-HRV-infections showed lower basal FEV1(%) than coinfections (*p* = 0.04) and lower z-score FEV_1_ than single-RSV-infections (*p* = 0.04) or coinfections (*p* = 0.02). Also, single-HRV-infections had lower post-bronchodilator FEV1(%) and z-score FEV_1_ values than coinfections (*p* = 0.03 and *p* = 0.03). Single-HRV-bronchiolitis was an independent risk factor for FEV_1_ < 80% (*p* = 0.007). FeNO value > 25 ppb was detected in 21(12.5%) cases, without differences between viral groups (*p* = 0.768). The prevalence of allergic sensitization was similar in coinfections (31.4%) versus single infections (38.7%), (*p* = 0.428). The highest frequency of allergic rhinitis was observed in single-HRV patients (*p* = 0.004). The respiratory morbidity at 7–9 years of coinfected patients was similar to the single-HRV ones. In contrast, the likelihood of current asthma was up to 5 times higher in RSV/HRV coinfections than in the single-RSV-infections ones (*p* = 0.012). The respiratory morbidity at 7–9 years of age after severe bronchiolitis is significantly higher in single-HRV or viral coinfection patients that in single-RSV ones. Single-HRV-bronchiolitis is independently associated with lower lung function at school-age.

Respiratory syncytial virus (RSV) causes up to 75% of bronchiolitis cases, but other agents, also associated with lower respiratory tract infection in this age group, include rhinovirus (HRV), human bocavirus (HBoV), human metapneumovirus (hMPV), influenza virus (FLU) or parainfluenza virus (PIV)^1–4^. Viral coinfections are relatively common in bronchiolitis, with a frequency that varies from 10 to 40% in hospitalized infants^5–7^.

It is well known that infants suffering from severe bronchiolitis are at increased risk of asthma development during childhood^8–11^. Asthma is one of the most common chronic respiratory diseases in children and several risk factors have been described, such as air pollution, passive smoking, genetic factors, early viral wheezing and others^12,13^.

Before the high frequency of rhinovirus detection in bronchiolitis was described, RSV was thought to be virtually the only viral infection that could predispose to the development of asthma in childhood. However, in the past few decades, HRV has been gradually recognized as a major pathogen causing acute wheezing in early life and HRV-induced severe bronchiolitis is currently considered a stronger marker of asthma risk than wheezing episodes caused by RSV^14–16^. In addition, other respiratory viruses have also been associated with higher risk of asthma development^17^. Therefore, the viral etiology of the first episode of severe bronchiolitis is becoming increasingly relevant when evaluating later outcomes in children with wheezing in early childhood. As mentioned above, two or more respiratory viruses are simultaneously identified in up to 40% of hospitalized children. Data on clinical severity in coinfections compared with single-infections during acute bronchiolitis are contradictory, with some studies suggesting greater severity in coinfections, whereas others do not find any significant differences between both groups^5–7^. Regarding the medium and long-term respiratory morbidity after bronchiolitis associated to viral coinfection, data are even scarcer. To our knowledge, only one study, previously published by our group, has analyzed the medium-term respiratory outcome in patients with previous severe viral coinfection bronchiolitis^18^. In that study, conducted by telephone interview, children with viral coinfection were 2.5-fold more likely to develop asthma at 6–8 years compared to those with single viral infection.

On the other hand, several studies have demonstrated that lung function abnormalities are present later in childhood, in adolescence and even in early adulthood after hospitalization for early-childhood wheezing^9–11,19^. However, there has been little standardization between studies, with contradictory results and most of them focused on RSV-bronchiolitis. To our knowledge, there are no published studies comparing school-age lung function in children hospitalized for bronchiolitis with simple viral infection versus viral coinfection.

The aim of the current study was to compare the lung function and the occurrence of atopy and asthma, at 7–9 years, in children previously hospitalized for bronchiolitis associated with viral coinfection *vs.* single viral infection.

## Methods

### Study design and subjects

This was an observational, longitudinal, post-bronchiolitis, hospital-based, follow-up study, that is a part of an ongoing prospective investigation of respiratory tract infections in children, approved by the Medical Ethics Committee. Written informed consent was obtained from all the parents/caregivers after full explanation of the study protocol. All methods were carried out in accordance with relevant guidelines and regulations.

**Figure 1:**
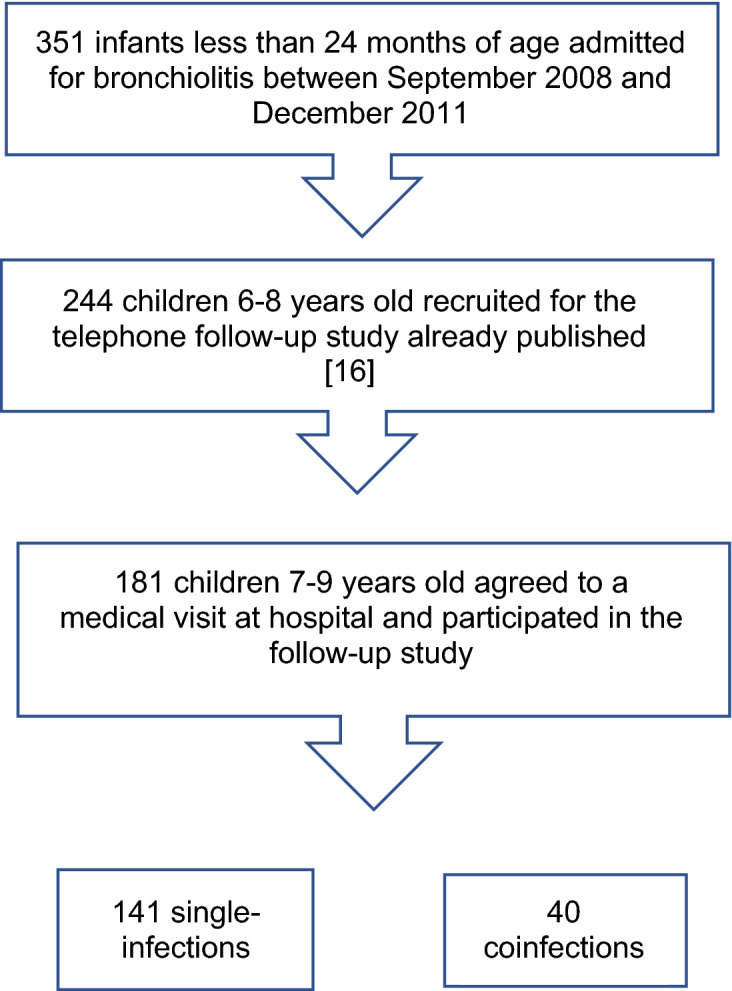
Flow chart of the subjects included in the study from the initial cohort to the follow‐up cohort.

Children below 2 years of age, hospitalized for their first episode of acute bronchiolitis in the Severo Ochoa University Hospital (Spain), between September 2008 and December 2011, were included (N = 351). A two-phase follow-up study was conducted. In the first one, whose results were already published, all previously included patients at admission were invited to a telephone follow-up visit, to compare the frequency of asthma in children with severe bronchiolitis associated with viral coinfection *vs*. single infection. A total of 244 children (52 coinfections and 192 single-infections) were located and agreed to participate^18^. In the current phase of the study, the aforementioned 244 children were invited to a face-to-face medical consultation at the hospital, including a clinical questionnaire, lung function assessment and skin prick test (SPT) for allergy Fig. 1.

### Clinical evaluation

The virological and clinical methods during the admission for bronchiolitis were previously published in detail^18^. At the follow-up visit, a structured questionnaire was used to obtain information on wheezing episodes, related hospital admissions, physician-diagnosed atopic dermatitis, allergic rhinitis, food allergy and use of bronchodilators and maintenance medication for asthma [inhaled corticosteroid, combination inhaled corticosteroid/long-acting beta-2 agonists (ICS/LABA), leukotriene antagonists]. Information on parental smoking habits, presence of allergy, eczema and asthma in first order family members (mother, father or siblings) previously diagnosed by a medical doctor was also recorded.

The ISAAC questionnaire for asthma symptoms for 6–7-year-old children, previously validated and translated to Spanish was also used in all cases^20^. *Current asthma* prevalence was estimated by the proportion of patients who responded affirmatively to the question number 2 of the ISAAC questionnaire (wheezing or whistling in the chest in the past 12 months), the one which has demonstrated the greatest correlation with current asthma prevalence in validation studies^21^. *Recurrent wheezing* was defined as the presence of wheezing episodes diagnosed by a doctor in the first 4 years of life^22^. *Current allergic rhinitis* was defined as present if at least two of the following three criteria were fulfilled: sneezing or a runny or blocked nose without having a cold; nasal allergy/hay fever medication, both in the past 12 months; doctor-diagnosed allergic rhinitis or hay fever ever^23^. *Atopy* was defined as a positive skin prick test (SPT) for at least one allergen.

### Fraction of exhaled nitric oxide (FeNO) measurement and lung function tests

FeNO was measured using the NIOX VERO handheld device, considering normal FeNO values < 25 ppb^24^.

Lung function was evaluated by forced spirometry, performed in accordance with the Spanish Society of Pulmonology and Thoracic Surgery (SEPAR)^25^. In order for the spirograms to be accepted, the second highest FEV_1_ and FVC had to lie within 0.15 L of the highest value, and the highest FEV_1_ and FVC were picked for the analyses. FEV_1_ /FVC and the mid-expiratory flow at 50% of FVC (MEF50) were taken from the spirogram with the highest sum of FEV_1_ and FVC. The flow-volume spirometry parameters were given as percentages of population-based, sex-specific, height related references, namely percentage of predicted^26^. The variables collected were: FVC (forced vital capacity), FEV_1_ (forced expiratory volume in one second), FEV_1_/FVC and FEF_25-75_ (mean expiratory flow between 25 and 75% of FVC). The results were values according to the reference values of The Global Lung Function Initiative (GLI) available at https://www.ers-education.org/guidelines/global-lung-function-initiative/about/^27^.

Spirometry values ​​were considered to be normal when FEV_1_ and FVC were ≥ 80%, FEV_1_/FVC > 90% and FEF25-75 ≥ 65% of their values^28^.

Post-bronchodilator test was considered positive when an increase of FEV_1_ of at least 12% compared to the baseline was observed after administration of 400 μg of inhaled salbutamol.

### Allergic sensitization

Allergic sensitization was evaluated by SPT to common aeroallergens, following the recommendations of the European Academy of Allergy and Clinical Immunology^29^. The following allergens were used: Dermatophagoides Pteronyssinus, Dermatophagoides Farinae, Alternaria Alternata, Cladosporium, grass, cypress, olive and platanus pollen, dog and cat dander.

Standardized extracts (ABELLO) were used, with a positive control (10 mg/ml histamine) and a negative one (glycerol saline vehicle solution). The test was considered positive when the wheal diameter was greater than or equal to that of histamine and 3 mm larger than the negative control.

### Statistical analysis

The sample size needed to detect a difference in FEV1(%) of at least 7 percentage points, with an alpha error of 5% and a power of 80%, was calculated. Estimating a rate coinfection/single infection of 1/3, 37 cases in the coinfection group and 111 in the single infection one would be needed.

Categorical variables were described using absolute and relative frequencies. Continuous variables were described using mean and standard deviation (normal distribution) or median and interquartile range (non-normal distribution).

Group comparisons were performed with Student’s t-test, Pearson’s chi-square test or Fisher’s exact test for tables with frequencies < 5, as appropriate. Data not normally distributed were analyzed by the Mann–Whitney U-test. Logistic regression models were constructed to assess a set of potential risk factors for current asthma. Each variable was entered separately into univariate models, and odds ratios (OR) with 95% CI were calculated. Explanatory factors with *p*-values < 0.2 in univariate analysis were further analyzed in a multiple regression model. *P*-value of < 0.05 was regarded as statistically significant. All analyses were two tailed, and were performed using the Statistical Package for the Social Sciences (SPSS), version 21.0.

### Ethics approval and consent to participate

The study was approved by the Ethics Committee of Severo Ochoa Hospital. Written informed consent was obtained from all the parents/caregivers after full explanation of the study protocol. All methods were carried out in accordance with relevant guidelines and regulations.

## Results

### Subjects

Of the 244 patients included in the first telephone-follow-up study, 181 (40 coinfections and 141 single infections) could be contacted again and agreed to a medical visit at hospital and constituted the population of the current study. Figure 1. The main reason for drop-out was a change in their telephone number. Children who lost follow-up did not differ significantly from others regarding initial hospitalization, gender, type of virus, prematurity, and age at inclusion.

All the 181 included patients completed the questionnaires, while 172 (95%) agreed to take part in SPT, and 177 (97.7%) agreed to perform lung function tests. Five children were not able to cooperate in the spirometry test and 14 were not able to perform the manoeuvre to measure FeNO, so that, 172 spirometries and 167 FeNO measurements were finally obtained.

### Clinical characteristics at admission for bronchiolitis

**Figure 2:**
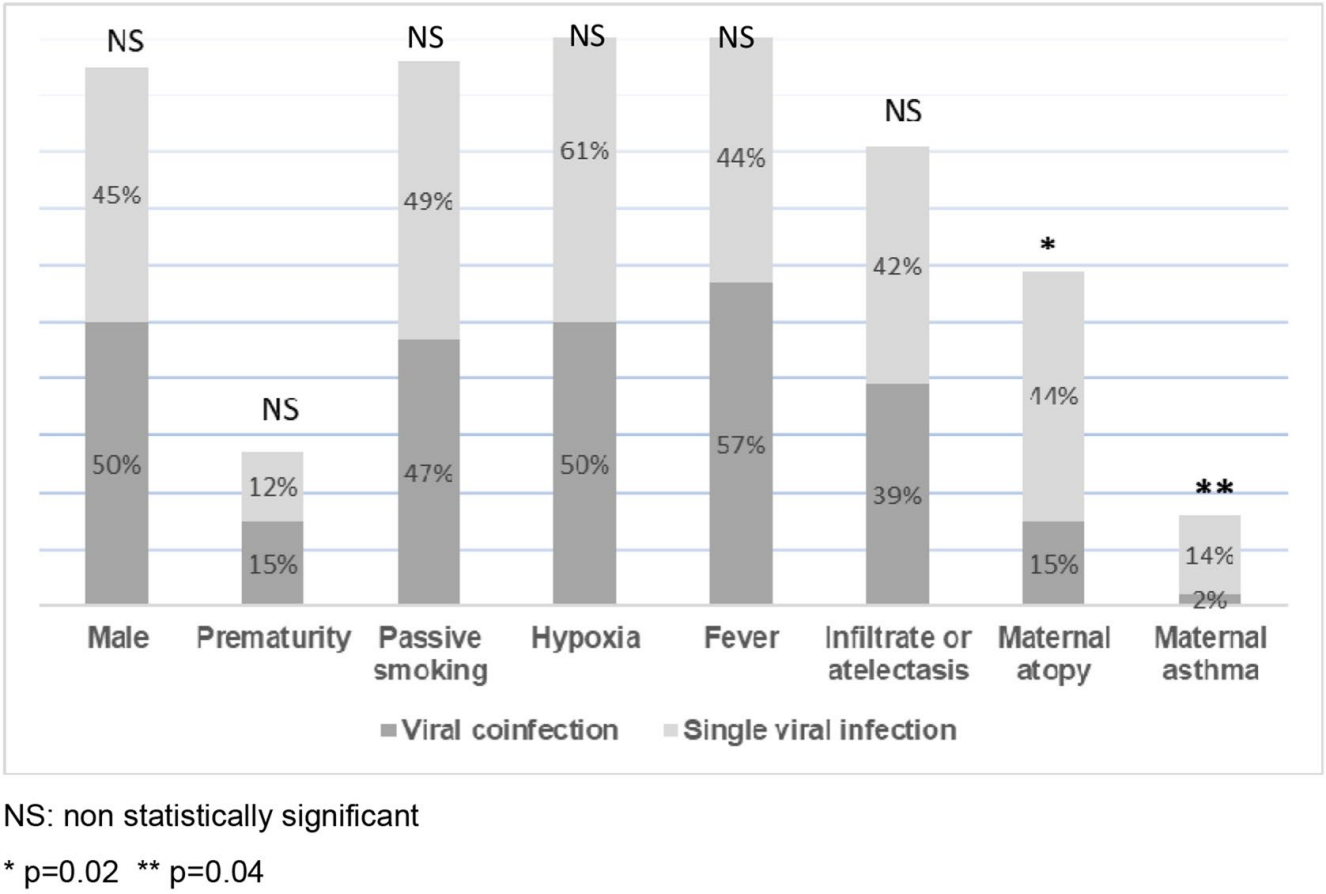
Clinical characteristics at admission of infants with bronchiolitis associated with viral coinfection versus single-viral infection (N=181).

Clinical characteristics during admission for bronchiolitis are presented in Fig. 2. No significant differences could be detected between both groups, with the exception of higher frequency of maternal asthma and atopy in the single-infection group (*p = *0.04 and *p = *0.02 respectively).

The most frequently identified viruses in single infections were RSV (70, 50%), HRV (29, 16%) and hMPV (8, 6%), whereas the most frequent viruses in coinfections were HRV (26, 65%), RSV (24, 60%) and HBoV (12, 30%). The most common associations were RSV + HRV (15, 29%) and RSV + HBoV (12, 23%).

### Comparison of respiratory morbidity at 7–9 years of children with single infections vs. viral coinfections

The median age at follow-up was 8.3 years (IQR: 7.5–9.1), similar in both groups. A high proportion of patients in both groups reported some episode of recurrent wheezing, 92% in the coinfection group and 89% in the single-infection group (*p = *0.483; OR: 1.58, CI95%: 0.44–5.71). Similar number of children in both groups needed rehospitalization for wheezing, 35% in coinfections and 36% in single infections (*p = *0.892), but the number of wheezing-related admissions was significantly higher in children with coinfections (3.6 ± 3.5 vs. 1.8 ± 1.7, *p = *0.005), who also reported higher frequency of respiratory symptoms in the intercrisis periods (11.4% vs. 3.4%, *p = *0.037; OR:4.16, CI95%: 0.98–17.57).

Current asthma at 7–9 years was independently associated to viral coinfection (*p = *0.004), allergic rhinitis (*p = *0.001), food allergy (*p = *0.05), positive SPT (*p = *0.04), atopic dermatitis (*p = *0.004) and preterm birth (*p* = 0.04). Table 1. Although paternal and maternal asthma were associated with current asthma in the univariate analysis, the association did not hold in the multivariate analysis.

**Table 1:** Bivariate and multivariate analysis of possible risk factors associated with current asthma treatment in the compiled coinfection and single-infection bronchiolitis groups.

Current Asthma	Bivariate Analysis			Multivariate Analysis		
	*P*- value	Crude Odds Ratio	Confidence interval 95%	*P*- value	Adjusted Odds Ratio	Confidence interval 95%
Viral Coinfection	0.09	1.69	0.91–3.13	0.004	3.20	1.40–6.90
Atopic Dermatitis	0.001	3.30	1.56–6.99	0.004	2.30	1.20–4.70
Allergic Rhinitis	< 0.001	3.97	1.93–8.18	0.001	3.50	1.70–7.20
Positive skin prick test	< 0.001	4.29	1.98–9.27	0.04	2.68	1.04–6.48
Food allergy	0.005	3.87	1.42–10.44	0.05	2.60	1.02–6.80
Premature birth	0.06	2.32	0.92–5.83	0.04	3.13	1.02–9.61
Maternal asthma	0.026	2.84	1.10–7.31			
Paternal asthma	0.05	2.36	0.98–5.70			

Both groups required chronic asthma treatment with a similar frequency (52% vs. 57.5%, *p = *0.141). However, the likelihood of receiving the combination inhaled corticosteroid/long-acting β2-agonist (ICS/LABA), indicated for a greater severity step, was 3.9 times higher in the coinfection group (15% vs. 4.3%, *p = *0.017). In the multivariate analysis, viral coinfection (*p* = 0.026), allergic rhinitis (*p < *0.001), maternal atopy (*p = *0.02) and passive smoking (*p* = 0.045), were independently associated with chronic asthma treatment requirement. Table 2.

**Table 2:** Bivariate and multivariate analysis of possible risk factors associated with chronic asthma treatment in the compiled coinfection and single-infection bronchiolitis groups.

	Bivariate analysis			Multivariate analysis		
	P-value	Crude Odds Ratio	Confidence interval 95%	P-value	Adjusted Odds Ratio	Confidence interval 95%
**Risk Factor for chronic asthma treatment**						
Viral Coinfection	0.09	1.69	0.91–3.13	0.026	2.11	1.1–4.08
Atopic Dermatitis	0.004	2.11	1.26–3.54			
Allergic Rhinitis	< 0.001	3.27	1.81–5.89	< 0.001	3.23	1.74–6.00
Environmental Tobacco Smoke	0.035	1.89	1.04–3.43	0.046	1.89	1.01–3.55
Maternal Atopy	0.007	2.26	1.24–4.10	0.022	2.10	1.11–3.96
Siblings Atopy	0.08	1.64	0.94–2.86			
**Risk Factor for Budesonide treatment**						
Atopic Dermatitis	0.008	2.10	1.20–3.65			
Allergic Rhinitis	< 0.001	2.92	1.61–5.27	0.002	2.64	1.44–4.86
Mother´s Atopy	0.001	2.69	1.46–4.95	0.01	2.29	1.22–4.31
Siblings´ Atopy	0.09	1.63	0.91–2.93			
Risk Factor for ICS/LABA* treatment						
Viral Coinfection	0.026	3.41	1.09–10.63	0.004	23.41	2.81–194.89
Infiltrate/atelectasis	0.05	3.64	0.88–15.08	0.027	10.01	1.31–74.66
Food Allergy	0.07	3.31	0.84–13.09			
Allergic Rhinitis	0.004	4.71	1.48–14.99	0.006	15.64	2.21–110.37
Siblings´ Asthma	< 0.001	8.37	2.62–26.71	0.003	21.55	2.93–158.64

* ICS/LABA: Inhaled corticosteroid/long-acting β2-agonist.

In addition to viral coinfection (*p = *0.004), the prescription of ICS/LABA was independently associated with allergic rhinitis (*p = *0.006), siblings´ asthma (*p = *0.003) and infiltrate/atelectasis during the admission for bronchiolitis (*p = *0.027).

After the global comparison between coinfections and single infections, single-RSV and single-HRV groups were compared with the coinfection one.

### Comparison of respiratory morbidity at 7–9 years of children with single-RSV and single-HRV vs. viral coinfections

Compared with single-RSV infections, children with viral coinfections needed significantly more chronic asthma treatment (*p = *0.05), montelukast (*p = *0.02) and ICS/LABA (*p = *0.005), presented more symptoms in the intercrisis periods (*p = *0.03), had been more frequently diagnosed with asthma ever and required higher number of admissions for asthma (*p = *0.03). Table 3. When single-RSV infections were specifically compared to RSV/HRV coinfections, the likelihood of current asthma was up to 5 times higher in coinfections (44% vs. 13%, *p = *0.012, OR:5.47, CI95%1.29- 23.25).

**Table 3:** Comparison of respiratory morbidity at 7–9 years of age in children with a history of bronchiolitis due to viral coinfection, single RSV infection and HRV infection.

	Viral coinfection (N = 40)	Single RSV infection (N = 70)	*P*- value^†^	Single-HRV infection (N = 23)	*P*-value^¥^
Recurrent wheezing	37(92.5%)	59 (84%)	0.214	23(100%)	0.178
Admissions for wheezing	14(35%)	20 (29%)	0.483	14(61%)	**0.04**
Number of admissions for wheezing	3.5 (3.5)	1.28 (1.2)	**0.03**	2.9 (1.9)	0.555
Symptoms in the intercrisis periods	4(11.4%)	1(1.5%)	**0.03**	1(4%)	0.108
Chronic asthma treatment	23(57.5%)	27(38.6%)	**0.05**	17(74%)	0.193
Budesonide	16 (40%)	24(34%)	0.549	13(56.5%)	0.205
Montelukast	19(47.5%)	18(25.7%)	**0.02**	15 (65%)	0.174
Salmeterol/fluticasone	6 (15%)	1 (1.4%)	**0.005**	1 (4%)	0.195

RSV: respiratory syncytial virus.

HRV: rhinovirus.

^**†**^Single- RSV infections compared to viral coinfections.

^**¥**^Single- HRV infections compared to viral coinfections.

Significant values are in bold.

In contrast, the respiratory morbidity at 7–9 years of coinfection-patients was quite similar to the single-HRV ones, with the exception of higher frequency of asthma admissions in children with coinfection (61% vs. 35%, *p = *0.045; OR:2.89, CI95%:1.10–8.33). Table 3.

When single-HRV infections were compared to single-RSV ones, higher frequency of current asthma was observed in HRV-patients (39% vs. 14.7%, *p = *0.01, OR:3.72, CI95%:1.27–10.90), as well as more asthma treatment (74% vs. 38%, *p = *0.003, OR:4.51, CI95%:1.58–12.87) and more admissions for asthma (61% vs. 29%, *p = *0.005, OR:3.89, CI95%:1.45–10.41). The association of single-HRV infections with current asthma remained statistically significant even in children without allergic sensitization (25% vs. 4%, *p = *0.028).

### Skin prick test and allergic rhinitis

Skin prick tests to common aeroallergens were performed on 172 children, of which 64 (37%) tested positive, mainly to outdoors allergens (grass pollen). Forty-six of them were polysensitized.

The prevalence of allergic sensitization in coinfections (31.4%) was similar to that of single infections (38.7%), (*p = *0.428). No differences were detected when coinfections were compared with single-HRV or single-RSV infections. On the other hand, atopy was significantly more frequent in children with current asthma (*p < *0.001, OR:4.29, CI95%:1.99–9.27), atopic dermatitis (*p = *0.02; OR: 2.10, CI95%:1.2–3.95), food allergy (*p = *0.001, OR: 6.0, CI95%: 1.84–19.52), allergic rhinitis (*p < *0.001, OR:9.54, CI95%:4.63–19.66), maternal asthma (*p = *0.01; OR:3.33, CI95%:1.23–8.96) and paternal atopy (*p = *0.05; OR:1.94, CI95%:0.97–3.87).

Overall, allergic rhinitis was diagnosed in 62(34%) cases, with the highest prevalence in children with single-HRV infection (52%), when compared to single-RSV (31%, *p = *0.07) or to coinfections (17%, *p = *0.004). We also found that 25 out of 42 children (60%) with current asthma had allergic rhinitis, compared with 37 out of 137 (27%) children without current asthma (*p < *0.001). And vice versa, 25 (40%) of the 62 children with allergic rhinitis had current asthma, compared with 14% of asthma in those without allergic rhinitis (*p < *0.001, OR:3.97; CI95%:1.93–8.18).

### Lung function

A total of 177 spirometries were performed, of which 172 were considered to be valid according to standard quality criteria. FEV_1_ < 80% was found in 15 cases (9%), 2 in the coinfection group and 13 in the single-infection one (54% of them were single-HRV). FEV_1_/FVC < 90% was observed in 16 cases (2 coinfections and 14 single infections).

FEV_1_ values, expressed as percentage, tended to be slightly higher in the coinfection group, although without reaching statistical significance (*p = *0.06). Patients with single-HRV infections showed lower FEV_1_ values in comparison to the coinfection ones (*p = *0.04). In contrast, no differences were found in FEV_1_ values between single-RSV infections and coinfections Table 4.

**Table 4:** Lung function at 7–9 years of age in children with history of severe bronchiolitis associated with viral coinfection versus single-viral infection.

	Viral coinfections (N = 40)	All single infections (N = 141)	*P* -value^†^	Single-RSV Infections (N = 70)	*P* -value^§^	Single-Rhinovirus infections (N = 23)	*P* -value^¥^
FEV_1_(% predicted)*	102.2 (14.4)	97.6 (13.2)	**0.06**	98.4 (11.5)	0.138	93.9 (15.1)	**0.04**
FEV_1_ z score*	0.14 (1.13)	−0.3 (1.1)	**0.04**	− 0.13 (0.95)	0.188	− 0.6 (1.1)	**0.03**
FVC (% predicted)*	98.2 (12.2)	96.6 (11.7)	0.456	96.3 (9.9)	0.400	92.9 (12.4)	0.118
FVC z score*	0.12 (1)	−0.04 (0.9)	0.357	0.02 (0.88)	0.604	− 0.46 (1.03)	**0.04**
FEV_1_/FVC (% predicted)*	104.3 (7,9)	101.9 (8.8)	0.148	103.2 (8.1)	0.508	101.6 (11.1)	0.284
FEV_1_/FVC z score *	0.1 0 (1.10)	−0.30 (1.20)	**0.05**	− 0.21(1.12)	0.227	− 0.20 (1.49)	0.438
FEF_25-75_ (% predicted)*	90.5 (23.1)	85.7 (25)	0.289	88.6 (24.6)	0.699	82.1 (28.5)	0.220
FEF_25-75_ z score*	−0.2 (1.1)	−0.5 (1.2)	0.108	− 0.30 (1.13)	0.507	− 0.69 (1.34)	**0.09**
FEV_1_ (% predicted)*	107.1 (14.1)	101.6 (14.7)	**0.04**	101.4 (12.9)	0.169	98.2 (16.0)	**0.03**
FEV_1_ z score post bronchodilator*	0.43 (1.18)	0.80 (1.18)	**0.109**	0.11(1.11)	0.169	− 0.28 (1.25)	**0.03**

* Mean ± standard deviation.

^**†**^All single- infections compared to viral coinfections.

^**§**^Single- RSV infections compared to viral coinfections.

^**¥**^Single- HRV infections compared to viral coinfections.

Significant values are in bold.

The z-score FEV_1_ values were higher in the coinfection group (*p = *0.04) compared to the single-infection one as a whole, but no differences were found when compared only with the single-RSV one (*p = *0.188). However, children with HRV-single infection showed lower z-score FEV_1_ values (-0.637 ± 1.187) than children with single-RSV (-0.131 ± 0.957, *p = *0.04) and with coinfection (*p = *0.03). Bronchopulmonary dysplasia and infiltrate/atelectasis during bronchiolitis were also associated with lower z-score FEV_1_ value (*p < *0.001 and *p = *0.04 respectively).

FEV_1_ values < 80% were observed more frequently in children with single-HRV- bronchiolitis than in single-RSV (*p < *0.001) or coinfections (*p = *0.006). The likelihood of having FEV_1_ values < 80% was 6 times higher in single-non-RSV infections than in single-RSV ones (3% vs. 17%, *p = *0.007, OR:6.60, CI95%:1.40–31.05). Bronchopulmonary dysplasia (*p < *0.001) and wheezing after exercising in the past 12 months were also associated with FEV_1_ < 80% (*p = *0.009) Table 5. Children with allergic rhinitis (*p = *0.663) or allergic sensitization (*p = *0.136) did not show higher frequency of FEV_1_ values < 80%. The variables associated with FEV_1_ < 80% in the bivariate analysis, with P-value < 0.20, were entered in a stepwise logistic regression analysis. Single-HRV bronchiolitis was the only risk factor independently associated with FEV_1_ values < 80% (*p = *0.023; OR:7.80, CI95%:1.33–45.63).

**Table 5:** Risk factors for FEV_1_ values < 80% in children 7–9 years of age, with history of severe bronchiolitis, viral coinfections and single-infections.

Risk factor for FEV_1_ < 80%	P- value	Odds Ratio	Confidence interval 95%
Viral coinfection	0.392	0.52	0.11–2.40
Single-Rhinovirus vs. Single-RSV infection	** < 0.001**	15.40	2.90–81.68
Single-Rhinovirus vs viral coinfection	**0.006**	8.40	1.56–45.20
Single-RSV vs viral coinfection	0.547	0.54	0.07–4.04
Allergic rhinitis	0.663	1.27	0.43–3.76
Atopy	0.136	0.38	0.10–1.41
Bronchopulmonary dysplasia	< 0.001	13.08	7.76–22.05
Wheezing after exercise past 12 months	0.009	4.09	1.32–12.62
Prematurity	0.080	2.86	0.81–10.04

Significant values are in bold.

The z-score FEV_1_/FVC value was also higher in the coinfection group (*p = *0.05) compared to the single infection one. Lower z-score FEV1/FVC values were more frequently found in children with bronchopulmonary dysplasia (*p* < 0.001), chronic asthma treatment (*p = *0.07), current asthma (*p = *0.016) and asthma ever (*p* = 0.03).

FEV_1_/FVC values ≤ 90% of predicted were observed more frequently in children with allergic rhinitis (*p = *0.015), chronic asthma treatment (*p = *0.025), treatment with budesonide (*p = *0.05), montelukast (*p = *0.009) and salmeterol/fluticasone (*p = *0.05), current asthma (*p = *0.01), wheezing after exercise (*p = *0.002) and night cough in the past 12 months (*p = *0.02). With respect to viral etiology, no differences between coinfection and single-infections were identified Table 6.

**Table 6:** Risk factors for FEV_1/_FVC values < 90% in children 7–9 years of age, with history of severe bronchiolitis, viral coinfections and single-infections.

Risk factor for FEV_1_ /FVC ≤ 90%	*P*- value	Odds Ratio	Confidence interval 95%
Viral coinfection	0.331	0.47	0.10–2.19
Single-HRV vs. Single RSV infection	0.660	1.60	0.27–9.39
Single-HRV vs. Single non-HRV infection	0.820	0.83	0.17–4.01
Single-HRV vs viral coinfection	0.567	1.80	0.23–13.77
Single-RSV vs viral coinfection	0.895	1.12	0.19–6.45
Allergic rhinitis	0.015	3.53	1.21–10.26
Chronic asthma treatment	0.025	4.01	1.01–14.63
Budesonide	0.050	2.74	0.95–7.93
Montelukast	0.009	4.31	1.33–13.97
Salmeterol/fluticasone	0.050	3.77	0.91–15.66
Current asthma	0.010	3.67	1.28–10.50
Wheezing after exercise past 12 months	0.002	5.00	1.68–14.86
Night cough past 12 months	0.020	3.47	1.14–10.47

HRV: Rhinovirus.

RSV: Respiratory syncytial virus.

Regarding the post-bronchodilator test, although no difference was found in the proportion of patients with positive test between coinfections and single infections (21.6% vs. 16.7%, *p* = 0.520), FEV1(%) values were higher in the coinfection group (107.1% ± 14.1 vs. 101.6 ± 14.7, *p* = 0.04), as well as FEV1/FVC values (106.5% ± 5.3 vs. 103.8 ± 8.5, *p* = 0.02). Children with single-HRV infections had lower post-bronchodilator FEV1 values than those with coinfections (98.2 ± 16.1 vs. 107.15 ± 1 4.11, *p* = 0.03), as well as lower z-score FEV1 values (− 0.28 ± 1.25 vs. 0.43 ± 1.18, *p* = 0.03). Conversely, no differences could be found between post bronchodilators values of children with single-RSV infections and those with coinfections.

The probability of positive bronchodilator tests was higher in children with current asthma (48% vs. 19.5%, *p* = 0.001).

To assess the possible confounding role of prematurity on lung function, FEV1, FVC and FEV1/FVC z-score values were compared between preterm and non-preterm children. No significant differences were found between them (Z-score FEV1: *p = *0.09; Z-score FVC: *p = *0.44 and Z-score FEV1/FVC: *p* = 0.065).

### FeNO

FeNO could be measured in 167 patients who were able to perform a valid test. Overall, 21 (12.5%) cases presented values > 25 ppb, without significant differences between children with coinfection (9.4%) and children with single infection (12.6%) (*p* = 0.768). No differences between single-RSV, single-HRV or coinfections were observed.

The proportion of children with FeNO > 25 ppb was significantly higher in those with atopy (*p < *0.001), atopic dermatitis (*p* = 0.019), allergic rhinitis (*p* = 0.003), history of admission for asthma (*p* = 0,02) and asthma ever (*p* = 0.004). Children with current asthma (*p* = 0.09) and wheezing after exercise in the past 12 months (*p* = 0.09) also showed a tendence to present higher levels of FeNO Table 7.

**Table 7:** Clinical features associated with fraction of exhaled nitric oxide (FeNO) > 25 ppb, at 7–9 years of age, in children with a history of bronchiolitis with single-viral infection and viral coinfection.

	FeNO > 25 ppb (N = 20)	FeNO ≤ 25 ppb (N = 147)	*P*- value
Viral Coinfection	3 (15%)	29 (20%)	0.614
Single-HRV infections vs coinfections	2 (40%)	20 (41%)	0.483
Single-RSV infections vs coinfections	8 (73%)	58 (67%)	0.686
Atopic dermatitis	15 (75%)	69 (47%)	**0.02**
Allergic rhinitis	13 (65%)	46 (31%)	**0.003**
Positive prick test	16 (84%)	45 (32%)	** < 0.001**
Asthma admissions	12 (60%)	50 (34%)	**0.024**
Current asthma	8 (40%)	33 (23%)	0.09
Asthma ever	8 (40%)	21(40%)	**0.004**
Wheezing after exercise in the past 12 months	6 (30%)	22 (15%)	0.09

RSV: respiratory syncytial virus.

HRV: rhinovirus.

Significant values are in bold.

## Discussion

To our knowledge, this is the first study to compare the lung function of school-age children with a history of severe bronchiolitis with viral coinfection *vs*. single-viral infection. Our results show that the coinfection group had slightly higher lung function values (FEV_1_, z-score FEV_1_ and z-score FEV1/FVC values) than the single-infection one, considered as a whole, at 7–9 years. However, when analyzing the different viral subgroups, children with coinfection had similar lung function to single RSV-infections and, both of them, had significantly higher values than single-HRV ones. The likelihood of having FEV1 < 80% was up to 15 times higher in the single-HRV group compared to the single-RSV one and up to 8 times higher than in the coinfection group.

Previous studies have analyzed the association between RSV-bronchiolitis and pulmonary function sequelae, although there has been little standardization between studies, with contradictory results^8–10,30–33^. A recent systematic review^34^ including 31 studies, found no association between RSV-infection during the first 3 years of life and abnormal pulmonary function in 13 studies, while 16 did reported this association. Many of the studies enrolled small numbers of participants, many of them used different pulmonary function techniques and reported different pulmonary function indices. But nevertheless, their results reflect varying abnormal pulmonary function when compared with infants without bronchiolitis, being the most commonly described, obstruction to airflow with or without bronchodilator reversibility. Most studies included compared pulmonary function in children with previously RSV-bronchiolitis with a control group of healthy infants. This is not the case in our study, in which the aim was to compare the lung function in children admitted for bronchiolitis with single *vs*. double or multiple viral infection and not *vs*. a control group of healthy children. We report here, for the first time, that children hospitalized for single-HRV bronchiolitis have significant worse lung function, at 7–9 years, than those admitted for bronchiolitis with viral coinfection or with single RSV-infection. Guilbert et al.^35^ in a longitudinal cohort of children at risk of asthma, prospectively explored the relationships among early life virus-specific wheezing, childhood lung function and asthma from 4 to 8 years of age, providing novel evidence that early HRV-wheezing illness, in high-atopy risk infants, are related to lower lung function in childhood, compared with children without HRV-wheezing illnesses. In contrast, children with RSV-wheezing did not have significant differences when compared with children who did not wheeze with RSV. Backman et al.^19^ noticed lower lung function tests in 14 cases with HRV- and 14 with RSV-induced early-childhood wheezing, compared to healthy controls, although without significant differences between the two viral wheezing groups. On the contrary, our results evidence that children with early single-HRV bronchiolitis have significantly lower FEV1 (%) and lower z-score FEV1 both pre- and post-bronchodilator than single-RSV or viral coinfections ones. Unlike Gilbert´s study^35^, which included a birth cohort of high-risk atopic children followed-up on an outpatient basis, with mostly mild respiratory infections in the first 3 years of age, all of our patients were hospitalized infants < 24 months of age and therefore, with more severe bronchiolitis, and without significant difference in the severity of the acute episode between coinfections and single infections. Therefore, the different respiratory morbidity observed at 7–9 years of age cannot be attributed, in our series, to greater or lesser severity of the acute bronchiolitis and might be related, among other factors, to the different viral etiology of the acute episode, suggesting than single-HRV early infections are associated with worse lung function development than single-RSV or coinfections.

Regarding to allergic rhinitis and atopy, our results show a global prevalence of allergic rhinitis of 34% in children previously hospitalized for bronchiolitis, considerably higher than the observed in 9623 6–7-year-old Spanish children in the GAN Phase I survey, with figures ranging from 12.5 to 18.8%^36^, or the 13.3% found in the prospective multicenter observational EuroPrevall-iFAAM birth cohort study, in 10 563 school-age children^23^. Our results, consistent with those of Ho et al.^37^, suggest that there is an association between bronchiolitis in infancy and allergic rhinitis later in childhood.

On the other hand, we also describe different prevalence of allergic rhinitis depending on the specific viral etiology of the acute bronchiolitis, with single-HRV infections having the highest rate of allergic rhinitis (52%) at 7–9 years of age, compared with single-RSV (31%, *p = *0.07) or with coinfections (17%, *p* = 0.004). It is worth noting that single-HRV patients had the highest rate of allergic rhinitis which, in turn, was associated with a four times higher probability of having asthma and lower lung function values.

The mechanism of the association of bronchiolitis, mainly that associated with single-HRV infection, with allergic rhinitis is not yet well known. Korppi et al.^38^ found that IL33 rs1342326 polymorphism was independently associated with allergic rhinitis and could also be associated with severe childhood asthma at school age. Our group, in a previous study ^39^, detected nasal IL-33 cytokine secretion in infants < 2 years of age hospitalized with bronchiolitis, with significantly higher detection rate in patients with HRV-infection. IL-33 plays an important role in T helper (Th) type 2 immunity, activating Th2-type lymphocytes, mast cells and eosinophils leading finally to allergic diseases such as allergic rhinitis^40^. It could be hypothesized that early viral lower respiratory infections, mainly HRV-infections, could enhance the release of different T2 cytokines, that could partly explain the higher rate of allergic rhinitis and current asthma found in single-HRV-bronchiolitis patients.

Studies on the prevalence of atopy among children with history of viral lower respiratory infection (LRTI) have shown contradictory results. Some studies found an increased risk of allergic sensitization^41^, other reported protection against allergic sensitization through the stimulation of Th-1 cytokine production^42^, whereas others did not find any influence of childhood viral LRTI on the risk of subsequent atopy^11^. The results of a recent systematic review and meta-analysis suggest, as ours, that there is no association between viral LRTI at < 5 years and positivity of skin prick test or atopic dermatitis, but, in contrast, there is a higher frequency of allergic rhinoconjunctivitis (OR = 1.7 [95%CI = 1.1–2.9])^43^.

The fraction of exhaled nitric oxide has been suggested as a non-invasive biomarker of eosinophilic inflammation^44^. Although in a recent study sedated single-breath FeNO, in wheezy infants/toddlers, was predictive of school-age asthma at age 6 years^45^ others^46^ found no differences in FeNO levels between 11-year-old children hospitalized for bronchiolitis and the control group. Prais et al. ^47^ did not find significant differences in FeNO in 8-year-old preterm children with and without palivizumab prophylaxis, suggesting that early RSV-infections are not associated with eosinophilic airway inflammation later in childhood. Similar results were described by Bar-Yoseph et al. ^48^. Our results showed no differences regarding FeNO levels between coinfection and single infection groups. By contrast, children with allergic sensitization had significantly higher FeNO values than not sensitized ones. No child with asthma but without allergic sensitization had elevated FeNO. These results, in line with those obtained by Mikalsen et al.^46^ suggest that only children who develop atopic asthma have eosinophilic airway inflammation, translated by elevated levels of FeNO.

According to our results, the probability of developing asthma at age 7–9 years was almost twice as high in children with coinfection (31%) than in those with single-infection considered as a whole (18%), being coinfection an independent risk factor for current asthma at school age. In addition, patients with viral coinfection showed greater respiratory morbidity, with higher rate of admissions for asthma, higher rate of treatment with the combination inhaled corticosteroid/long-acting β2-agonist (ICS/LABA), usually prescribed for moderate/severe asthma, and persistence of symptoms in the intercrisis periods. All these data strongly suggest that coinfections seem to be associated with increased respiratory morbidity, at least until the age of 7–9 years, when compared with single-infections. However, as was observed for pulmonary function, when coinfections were compared only with single-HRV infections, no significant differences were found in terms of frequency of current asthma or chronic anti-asthma treatment. In contrast, the frequency of current asthma in single-RSV children was 5 times lower than in children with coinfection and 4 times lower than in children with single-HRV infection. Our data suggest that bronchiolitis associated with single-HRV or viral coinfection pose a significantly higher risk, not only for the development of asthma, but also for more severe asthma, than single-RSV bronchiolitis.

Hyvärinen et al.^49^ reported in 2005 for the first time, the link from HRV-induced wheezing in infancy to emergence of childhood asthma. Since then, the role of HRV in bronchiolitis and the subsequent development of recurrent wheezing and asthma has been described^50,51^ and this association still remained significant in children ≥ 10 years^52^. Recently, Hasegawa et al.^53^ found that the risk of developing recurrent wheeze differed by the causative virus of the bronchiolitis, with higher risk for children with rhinovirus C infection compared to RSV. As previously mentioned, we could not compare the role of different types of HRV because our sample size was not calculated to achieve this goal. On the other hand, Lukkarinen et al.^54^, in a follow up study in 127 children, found that HRV-induced first wheezing episode predicts atopic but not nonatopic asthma at school age. In contrast, in our cohort, single-HRV bronchiolitis remained associated with asthma even in children without allergic sensitization.

Our study has potential limitations. Firstly, we did not have information from healthy controls because our objective was to compare the role of different viral etiologies in early infancy on the development of asthma, but not to compare it with healthy children. Also, the sample size was calculated to compare the lung function at 7–9 years in children previously admitted for bronchiolitis with single infection or coinfection and therefore, some of our findings, not related with this goal, should be confirmed in larger studies with enough statistical power. On the other hand, the number of single infections with viruses other than RSV or HRV is not very large, due to the high rate of coinfections among some viruses like HBoV. Finally, our results cannot be extrapolated to mild respiratory infections in an outpatient setting, as all of our patients needed hospital admission. That is, the respiratory morbidity observed in our series only reflect the evolution observed in severely affected children with need of hospital admission.

The strengths of the study are the prospective design and longitudinal follow-up. The study population is fairly homogeneous as only hospitalized patients with bronchiolitis younger than 2 years were included. At admission, RT-PCR to identify 16 respiratory viruses were performed in nasopharyngeal aspirate and clinical data were prospectively recorded. At follow-up, a standardized and validated clinical questionnaire (ISAAC questionnaire) was used and lung function as well as FeNO measurement could be performed in a high percentage of patients. On the other hand, specific tests (SPT) were done to confirm allergic sensitization.

In summary, according to our results, the respiratory morbidity at 7–9 years after severe bronchiolitis associated with viral coinfection or single-HRV infection is significantly higher than with single-RSV infections. Single-HRV severe bronchiolitis is independently associated with lower lung function values. Identification and follow-up of young infants hospitalized with viral coinfections and HRV-bronchiolitis seems to be crucial to indicate therapeutic and prevention strategies to improve their respiratory evolution.

## Data Availability

The datasets used and/or analysed during the current study are available from the corresponding author on reasonable request.
